# Intraspecific Trait Variation in the Mammalian Gastrointestinal Tract: A Digestive Dive and Systematic Literature Review

**DOI:** 10.1093/icb/icag070

**Published:** 2026-06-08

**Authors:** Olivia S Chapman, Bryan S McLean

**Affiliations:** Department of Biology, University of North Carolina at Greensboro, 312 Eberhart Building, 321 McIver Street, Greensboro, NC 27412, United States; Department of Biology, University of North Carolina at Greensboro, 312 Eberhart Building, 321 McIver Street, Greensboro, NC 27412, United States

## Abstract

The gastrointestinal tract (GIT) represents the functional link between food and energy for organisms. It also harbors the gut microbiome, protects against toxins, and eliminates waste, all of which mediate individual health and fitness. The form and function of the GIT can be dynamic, allowing organisms to respond to—and buffer against—rapid changes in their environment and energetic demands. However, knowledge of intraspecific trait variation (ITV) in the GIT is sparse among different taxa and traits (e.g., microstructural traits and physiology). Additionally, the actual mechanics of how these changes occur (e.g., cell proliferation, cellular redistribution) remain largely unknown. This systematic review summarizes the current state of knowledge on mammalian GIT ITV at multiple levels (macroscopic, microstructural, and physiological), speculates as to how global change drivers will affect ITV, and suggests new directions for future work. A comprehensive list of mammal species heretofore examined for GIT morphology and ITV from a total of 260 journal articles or book chapters identified 824 mammal species with quantitative GIT traits available (12% of all mammal species), but only 79 species (1.1%) investigated for GIT ITV. This highlights the increased need to preserve and collect trait data in standardized ways for mammalian GITs, a series of organs typically discarded or unused during specimen preparation. Understanding how wild species utilize GIT ITV to cope with energetic costs will be crucial to predicting how species may fare under rapidly changing environments.

## Introduction

The gastrointestinal tract (hereafter; GIT) is a highly sensitive organ system that can vary in response to changing energy availability (e.g., diet) or energetic demands (e.g., reproduction, cold exposure, parasitic infections). A dynamic GIT is hypothesized to provide capacity to buffer against—and quickly respond to—changes in available food resources or the energetic costs of essential physiological and life history processes. GIT variation below the species level has been documented in a variety of vertebrate taxa, including mammals (Naya, Karasov and Bozinovic 2007; Naya 2008), fish (Olsson et al. 2007; Zaldúa and Naya 2014), reptiles (Holmberg et al. 2003; Naya et al. 2011), amphibians (Naya and Bozinovic 2004; Relyea and Auld 2004), and birds (Sabat et al. 1998; Lv et al. 2014). Nevertheless, knowledge of how most taxa employ GIT modifications in response to a diversity of possible stimuli is minimal.

Intraspecific trait variation (hereafter; ITV) is broadly defined as the total amount of variation existing for any given trait within individuals of the same species (Moran, Hartig, and Bell 2016). ITV can be the result of heritable variations as well as phenotypic plasticity (the ability of a single genotype to express multiple phenotypes as a result of changing environmental conditions; West-Eberhard 1989; Henn et al. 2018). Plasticity is considered one of the main drivers of ITV (Mitchell and Bakker 2014; King et al. 2018), and the gold standard methods for demonstrating plasticity are longitudinal studies (tracking the same individual through time) or reaction norm experiments (genetically identical organisms used to test the effects of various environmental factors). However, collecting GIT traits often requires euthanasia of an individual, making it impossible to track the same individual across time. While live imaging and other advancements make it possible to measure internal structures of a living individual (e.g., X-ray images to obtain skull measurements of anesthetized shrews; Lázaro et al. 2017), it remains to be seen if this can be accurately applied to gut measurements of live animals. We thus use the term “ITV” in this review, but acknowledge that many of the patterns observed may reflect some amount of true plasticity.

Within mammals, the weight of studies to date linking GIT morphology and environmental conditions suggest some generality in how ITV emerges across space and taxa. For example, a correlation exists between GIT ITV and latitude in rodents, with higher levels of flexibility seen in higher latitude, more seasonal environments (Naya et al. 2008; Naya et al. 2014). Similar patterns have been documented in response to increasing elevation (Hammond et al. 1999). Future global changes may be expected to alter a number of these patterns. For example, consequences of climate change may include range shifts to higher latitudes and elevations (Johnston, Freund, and Schmitz 2012; Hetem et al. 2014), as well as altered reproductive cycles and shifts in the quality and quantity of food available across the year. Changing temperatures can also cause temporal mismatches between the timing of food availability and key processes such as hibernation (Kucheravy et al. 2021; Chmura et al. 2023). Additionally, movement to novel environments may impact parasite encounter rates and infection. Understanding how ITV is utilized as a response to these stressors and to buffer against changing climates and environments is crucial to understanding how species will maintain energetic balance under changing conditions.

In this review we aim to

1. Quantify the number of mammal species for which GIT ITV has been documented, both across the mammalian tree of life and between natural and laboratory settings.
2. Summarize the current knowledge of mammalian GIT ITV as a response to six main stressors (reproduction, cold exposure, dietary quality, hibernation/starvation/fasting, parasitic infection, and seasonality).
3. Consider the role that global change may have on the intensity of these stressors, thus indirectly affecting GIT ITV.
4. Suggest promising new directions for mammalian GIT ITV research.

## Methods

**Fig. 1:**
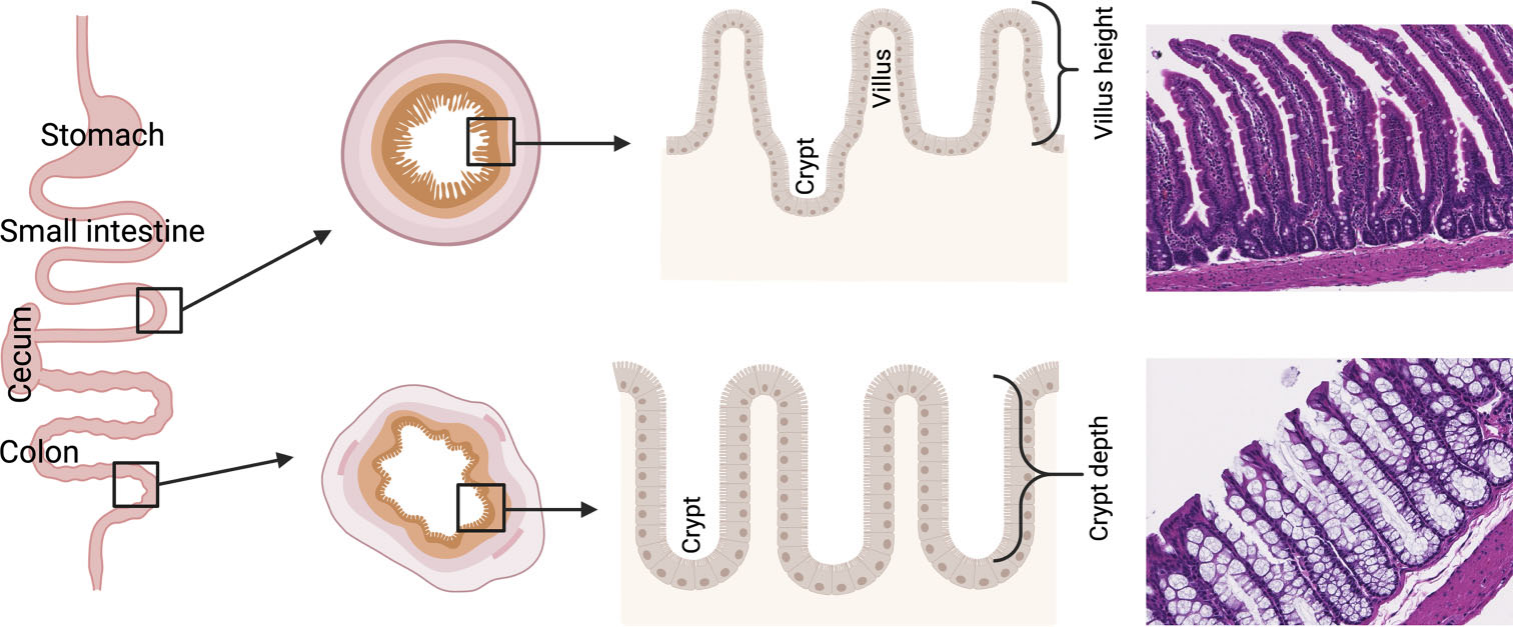
Schematic showing select macroscopic and microstructural traits of a mammalian gastrointestinal tract (note that some species lack a cecum). The large intestine is comprised of the cecum, colon, rectum, and anus. A cross section of the small intestine shows villi with crypts in between, while a cross section of the colon shows that only crypts are present. Macroscopic traits from this literature review included length, relative length (the length scaled to the individual’s body size), wet mass (with and/or without contents), and dry mass of one or more sections. Microstructural traits included villus height and/or width, villus density, crypt depth and width, density of goblet and Paneth cells, mucosal and serosal dry mass, and thickness of different layers of the GIT. We also recorded if physiological traits were taken, which include digestive enzyme activity, retention time and microbiota composition. Created with BioRender.com.

**Fig. 2:**
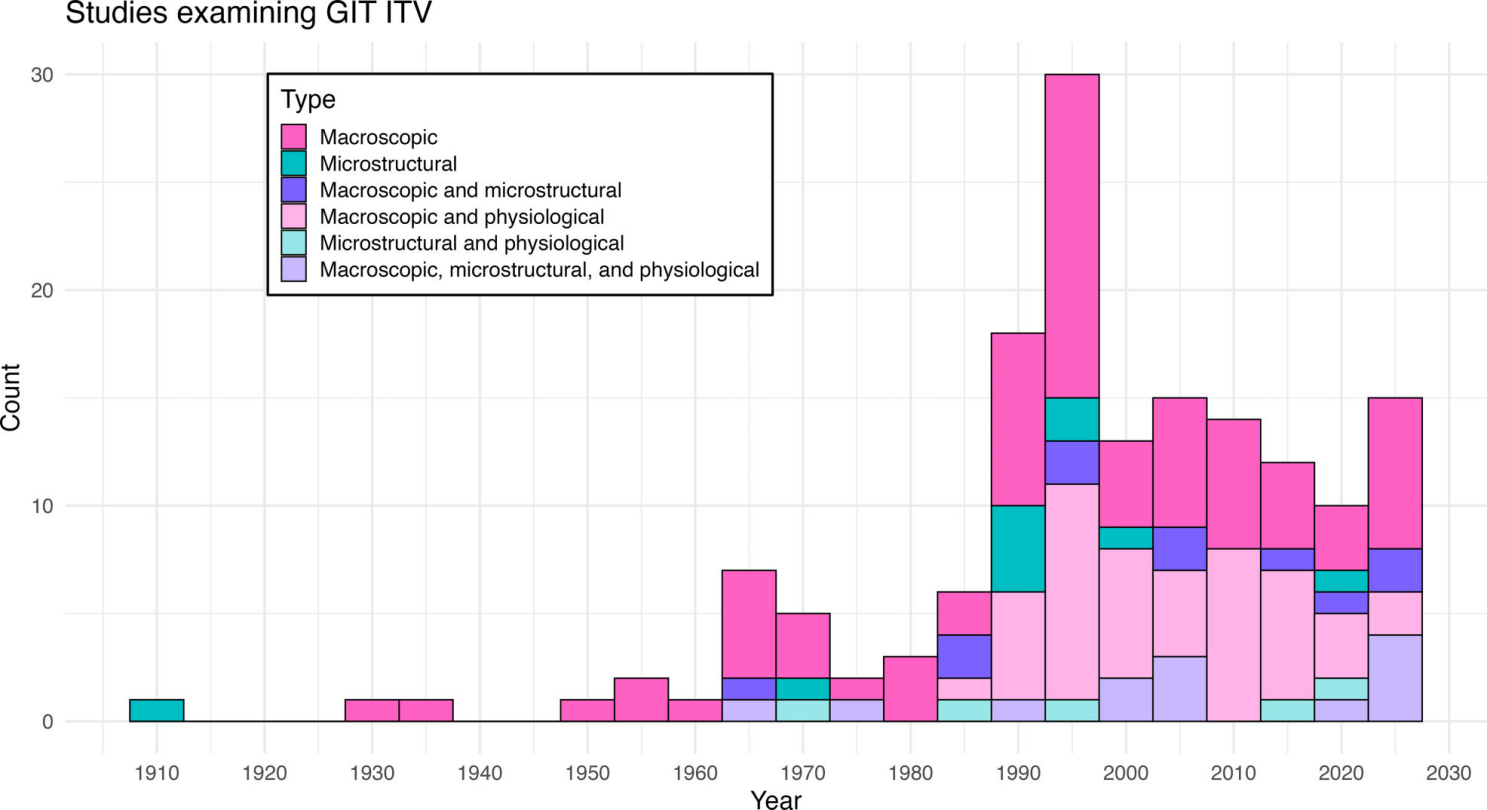
Frequency of papers in our dataset published each 5-year interval exploring gastrointestinal tract ITV in mammal species. Bars are colored by what type of ITV an individual study included (macroscopic, microstructural, physiological, or a combination of those three).

We used Google Scholar (https://scholar.google.com/) to perform a literature search for all papers published through December 31, 2025. We searched using the phrases “mammal intestine measurements,” “mammal gastrointestinal morphology,” “small mammal gastrointestinal plasticity,” and “mammal gut morphology.” We located additional sources via a comprehensive review of bibliographies. We only included sources that were focused on mammals and reported a quantitative measurement for at least one section of the GIT (stomach, small intestine, large intestine). For macroscopic morphology, this included studies of section length, relative length (the length scaled to the individual’s body size), wet mass (with and/or without contents), and dry mass. For microstructural morphology, we included studies of villus height and/or width, villus density, crypt depth and width, density of goblet and Paneth cells, mucosal and serosal dry mass, and thickness of different layers of the GIT (Fig. 1). If other aspects were measured (e.g., digestive enzyme activity, basal surface area, luminal surface area, nominal surface area, mucosal surface area, retention time, microbiota composition, volume of GIT sections), these were also recorded. We mapped all species names to the taxonomy of the Mammal Diversity Database v2.4 (https://www.mammaldiversity.org/;Burgin et al. 2025). A total of 260 journal articles or book chapters with these mammalian GIT measurements were identified; of these, 157 records dating back over 100 years focused specifically on ITV (Fig. 2). One author (OSC) screened all records to determine if they met our inclusion criteria. We have provided a Preferred Reporting Items for Systematic Reviews and Meta-Analyses (PRISMA) 2020 checklist as Supplementary Material 1, which was assembled after our initial literature review, in order to increase the transparency of our process (Page et al. 2021).

For each record that met our criteria above, we also recorded whether the individuals measured were wild-caught, reared in the lab, wild-caught and maintained in the lab, or captive/domestic. We recorded original measurements and retained the same terminology used by prior authors (e.g., many authors use colon and large intestine interchangeably, although these technically differ). Additionally, we did not remove duplicates from our metadata; for example, if a species appeared in prior compilations (e.g., Duque-Correa et al. 2021), we listed that as well as the original publication data was collected from. The full metadata can be found in Supplementary Material 2.

**Fig. 3:**
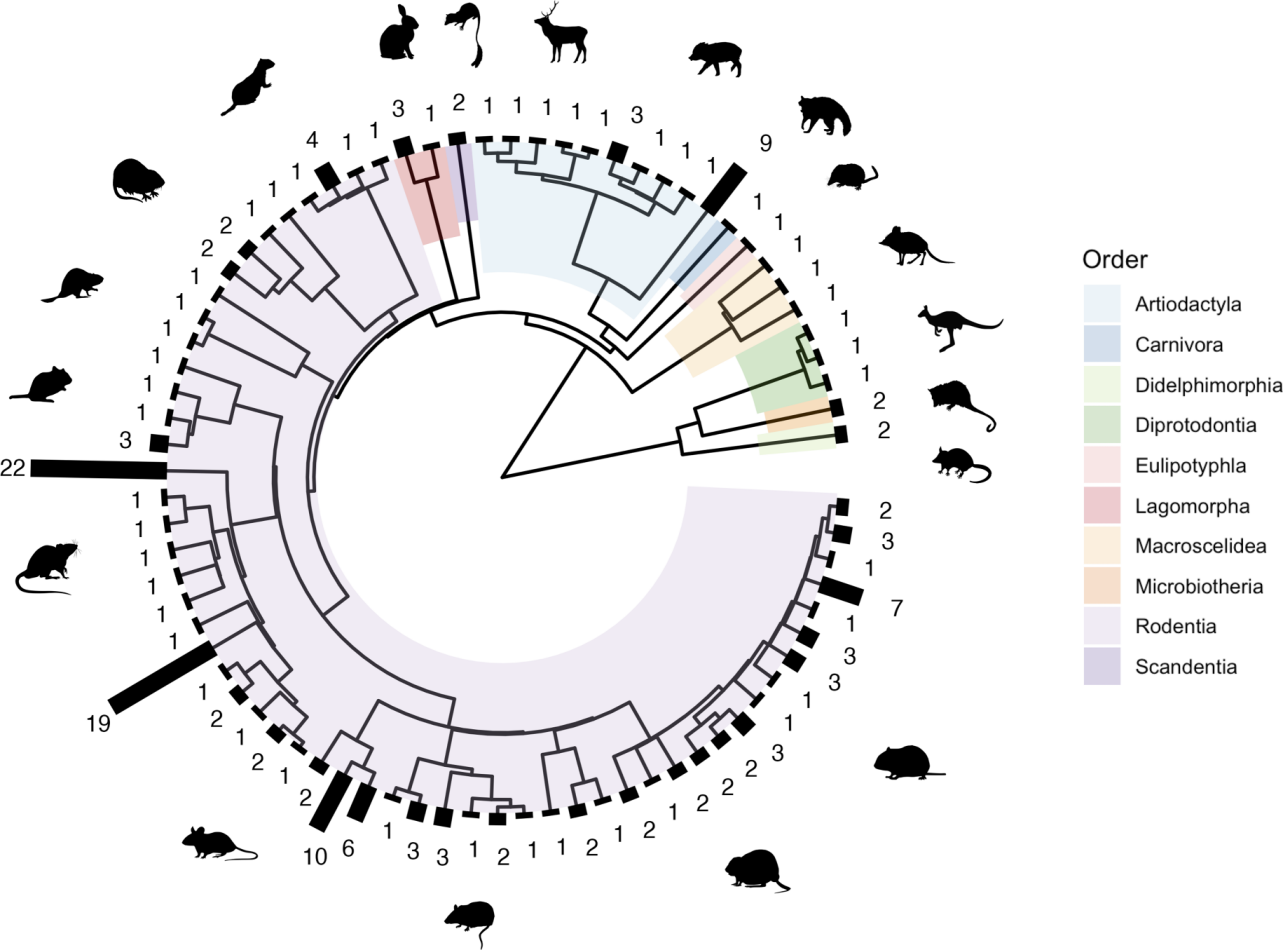
Phylogeny of mammals pruned to those species studied in the context of gastrointestinal tract intraspecific variation, colored by order. Black bars represent the number of studies done on each species.

We mapped summaries of the resulting data onto the mammal phylogeny from Upham, Esselstyn, and Jetz (2019) pruned to include all mammal species with quantitative GIT measurements (Supplementary Fig. S1), as well as all mammal species studied for GIT ITV (Fig. 3). Some species in Supplementary Material 2 were not present in this mammal phylogeny, so are not included in our final figures.

### Reproduction

Class Mammalia is split into three major groups based in part on reproductive strategies. Monotremes (Order Monotremata) lay eggs, marsupials (Infraclass Marsupialia) give birth to underdeveloped young that finishing developing in their mother’s pouch, and placentals (Infraclass Placentalia) have longer periods of gestation in order to give birth to well-developed young (Edwards and Deakin 2013; Ferner, Schultz, and Zeller 2017). ITV induced by reproduction has been observed in some or all sections of GITs from four placental Orders (Artiodactyla, Carnivora, Eulipotyphla, and Rodentia; e.g., Abramson 1934; Smith and Baldwin 1974; Derting 1996; Jaroszewska and Wilczyńska 2006). Given that mammals invest substantial energetic resources in rearing their young, reproduction is one of the most well-studied processes inducing morphological changes in the mammalian GIT. Lactation (production of milk from mammary glands) is the most energetically costly phase of reproduction for mammals, while gestation requires energy at a lesser extent. It has been estimated that a lactating female northern short-tailed shrew (*Blarina brevicauda*) weighing 11 g must produce enough milk to support 55 g of offspring (Pearson 1944). However, the relative investment in different reproductive phases varies among major clades; marsupials have longer periods of lactation and thus generally invest more energy into milk production than monotremes and placental mammals (Stannard, Miller, and Old 2020). Different reproductive phases are therefore expected to induce a different magnitude of GIT change depending on clade, although cross-clade comparisons are extremely rare.

A changing climate is likely to alter the timing of—and total investment in—reproduction for some mammals (Adams 2010; Zhao et al. 2020). In species capable of breeding multiple times per year (polyestry), a warming climate could create a longer breeding window, possibly resulting in more—but smaller—litters on average, reducing the energetic burden of single reproductive events (Tafani et al. 2013). Smaller litter sizes, and thus a smaller investment in lactation, could potentially require less GIT flexibility but only if available resources at the time of breeding remain stable.

However, in actuality, climate shifts and other global changes can lead to trophic mismatch, altered food availability, or shorter resource pulses, each of which could create a need for greater ITV to support reproduction. For example, increases in the number of breeding attempts per year in polyestrous species (e.g., due to more frequent failed attempts) may require sustaining a larger GIT for a higher proportion of the year. Additionally, a high body condition (i.e., substantial fat reserves) is needed in many species for a female to successfully reproduce (Lewis and Kappeler 2005; Michel and Bonnet 2012); increased GIT ITV may therefore be required in some years to offset poor body condition or the risk of producing no litter at all, especially in monoestrus species. More work is required to understand how potential consequences of climate and resource-related change will affect number of reproductive attempts and long-term fitness of mammals.

### Macroscopic ITV

Reproduction triggers changes in both the length and mass of the GIT in many mammals. Because small mammals on average have higher metabolic rates and larger litter sizes relative to large mammals, the GIT morphological response to reproduction is often exaggerated in the former (Hammond 1997). For example, lactation requires over a 400% increase in energy intake (Hammond 1997) in some rodents and involves direct costs such as organ remodeling, and indirect costs such as reductions in thermoregulation and immunocompetence (Speakman 2008). Lactation can also result in hypertrophy of the gut in some rodents (Abramson 1934; Craft 1970; Hammond and Diamond 1992; Naya et al. 2008), although this often reverses over the course of weaning. Pregnancy is not as energetically costly as lactation in placentals (Gittleman and Thompson 1988; Clutton-Brock, Albon, and Guinness 1989; Speakman 2008) and thus has a lesser impact on GIT morphology of rodents, but increases in GIT length and mass have still been shown during pregnancy in mice and rats (Barnett and Widdowson 1971; Cripps and William 1975; Speakman and McQueenie 1996). As expected, the magnitude of GIT flexibility has been positively correlated with litter size (Hammond and Diamond 1994; Zhang et al. 2016), as larger litters create more of an energetic strain for the mother (Norrie and Millar 1990).

The effect of reproductive investments on GIT ITV of males has been less commonly studied, but gut sections of some rodents are shorter and/or lighter in reproductively active males than their nonreproductively active counterparts (Chapman and McLean 2023; Hauksdóttir 2025). This inverse pattern is possibly a consequence of spending less time foraging or that the breeding season is otherwise less energetically challenging than winter. Thus, in general, females tend to display higher reproduction-related gut macroscopic ITV than males of the same species, with change being highest during the lactation period, although more research is needed outside of Placentalia. Global change-induced shifts in reproductive timing and litter size are therefore one factor that may induce changes in gut macrostructure.

### Microstructural ITV

Ultimately, some macroscopic changes in GITs (e.g., mass) derive from changes in the microstructure, especially mucosal thickness and villus and crypt dimensions. Reproduction can alter GIT microstructure in these ways. Villi are fingerlike projections that line the small intestine (Fig. 1; hereafter, SI) and increase absorptive area and capacity (Campbell, Berry, and Liang 2019). Crypts (Fig. 1; located between villi) dip down into the intestinal mucosa and contain stem cells that become epithelial cells, making them essential to the maintenance of villi (Bass and Wershil 2018). No villi exist in the colon, but crypts are still found throughout its length. Several studies confirm that lactation in mice and rats is associated with more complex crypts (Fell, Smith, and Campbell 1963), taller and wider villi (Fell, Smith, and Campbell 1963; Campbell and Fell 1964; Craft 1970), and increased mucosal thickness (Hammond and Diamond 1992). Current work is largely limited to rodents but suggests that lactation increases the robustness of the gut microstructure to compensate for the severe jump in energetic demand.

### Physiological ITV

An exciting but underexplored avenue of GIT research is functional ITV, particularly changes in nutrient uptake and the activity of different digestive enzymes. Increases in energetic demand would be expected to result in increased digestive activity and nutrient uptake, but the extent to which these changes in function are utilized alongside changes in form versus function has rarely been investigated. Lactation increases uptake of glucose and proline (an amino acid crucial to the repair and maintenance of the intestinal lining and a building block of collagen) in mice (Hammond and Diamond 1992), although no change in uptake was seen with increased litter sizes (Hammond and Diamond 1994). A study on captive hamsters (*Cricetulus barabensis*) found that lactation caused a significant increase in the activity of stomach pepsin and SI maltase, sucrase, and aminopeptidase-N activity, and that these increases were positively correlated with litter size (Zhang et al. 2016). Pepsin and aminopeptidase-N are necessary to break protein into absorbable units, while maltase and sucrase break down maltose and sucrose into absorbable glucose and fructose, respectively. These studies suggest that reproduction also triggers physiological changes in the GIT, but more work is needed to uncover the impacts of these changes on macroscopic and microstructural morphology of the gut.

### Cold exposure

Intraspecific trait variation as a response to decreased ambient temperature has been observed in some or all GIT sections in three of 27 mammalian Orders (Didelphimorphia, Rodentia, and Scandentia; e.g., Nespolo et al. 2002; Zhu, Zhang, and Wang 2012; Eto et al. 2016), and is particularly well studied in laboratory populations of small mammals. Small mammals have proportionately higher metabolic rates, partly due to thermoregulatory demands and a high surface area to volume ratio. As a body size guild, they employ various strategies to maintain energetic balance including increasing (Phillips and Heath 1995) or temporarily decreasing (via torpor or hibernation; Ruf and Geiser 2015) their metabolic rates and thus body temperatures. GIT form and function are intimately related to all of these temperature regulation factors. Winter conditions can be disproportionately impacted by climate change, particularly at high latitudes (Kreyling 2010; Williams, Henry, and Sinclair 2015), where winters are becoming milder and snow cover is declining in many places (Bormann et al. 2018). Warming winter temperatures may decrease the magnitude of seasonal ITV by curtailing the most extreme changes associated with coldest seasons, but it is unknown whether this can operate independently of the response to changes in some food resources that also occur seasonally.

### Macroscopic ITV

A multitude of studies has examined the impacts of cold exposure on the GITs of rodents in laboratory settings. Most studies find that that maintaining animals at lower temperatures results in increased length and mass of some or all gut sections (Heroux and Gridgeman 1958; Barnett and Widdowson 1965; Hammond and Wunder 1995; Hayword, Robertson, and McClelland 2022). The SI, which is the longest section of the GIT, seems to be the most prone to increases in length and mass when animals are maintained at lower temperatures, followed by the cecum. The impacts of cold exposure have also been examined in Northern treeshrews (*Tupaia belangeri*; Order Scandentia) and elegant fat-tailed opossums (*Thylamys elegans*; Order Didelphimorphia) with similar findings as in rodents; SI length and SI and stomach masses were greater (Zhu, Zhang, and Wang 2012) and cecum mass was greater (Nespolo et al. 2002) in these species maintained at lower temperatures, suggesting that milder winters and/or increasing ambient temperatures by themselves may decrease the overall cyclical GIT ITV in high-latitude mammals.

### Microstructural ITV

The effects of temperature on gut microstructure are less well known than for macroscopic morphology (length and mass). However, a few studies on captive populations of both New World (eastern deer mice; *Peromyscus maniculatus*) and Old World (house mice; *Mus musculus*) mice found that cold exposure led to an increase in small intestinal internal surface area (area integrated over the SI length; Toloza, Lam, and Diamond 1991). Compounded effects of cold exposure and other energetic stressors (pesticide exposure, French and Porter 1994; lactation, Hammond and Kristan 2000) resulted in higher mucosal mass in the SI, suggesting that cold exposure induces microstructural changes that underlie some changes seen in the macroscopic morphology of the gut.

### Physiological ITV

Cold exposure can increase energy assimilation and digestive efficiency in mammals, presumably as a plastic change to increase survival during winter months (Bozinovic and Nespolo 1997). This includes changes in nutrient uptake and enzyme activity. For example, acclimation to cold temperatures has been shown to increase levels of proline uptake in the SI of *M. musculus* (Konarzewski and Diamond 1994). Cold also increased small intestinal sucrase levels in Azara’s grass mice (*Akodon azarae*; del Valle, López Mañanes, and Busch 2004) and aminopeptidase-N levels in *T. elegans* (Nespolo et al. 2002), indicating that cold exposure induces increases in the activity of various digestive enzymes. As for reproduction, more integrative work is needed to study temperature-driven physiological changes in concert with morphological ITV of the gut.

### Dietary quality

GIT ITV in response to altered feeding regimes or diet quality has been observed in some or all sections of GITs of placentals and marsupials from eight mammalian Orders (Artiodactyla, Didelphimorphia, Diprotodontia, Lagomorpha, Macroscelidea, Microbiotheria, Rodentia, and Scandentia, e.g., Smith, Hubartt, and Shoemaker 1980; Sabat, Bozinovic, and Zambrano 1995; Spinks and Perrin 1995; Munn et al. 2009; Cortés et. al 2011; Finotti, Santos, and Cerqueira 2012; Gao et al. 2016; Tahas et al. 2017), making it the most well-studied stressor we found in this review. Global change factors have altered plant and insect phenology, which can result in shorter or earlier pulses of resources that many mammals depend on (Brown et al. 2022). Shifting geographic ranges, decreased species diversity, and increased presence of invasives can potentially alter the quality of forage available to herbivorous mammals. Insect biodiversity and abundance is also rapidly decreasing in some parts of the globe (Forister, Pelton, and Black 2019; Goulson 2019) and, while studies of dietary change related to insect abundances are scarce, this is a potential constraint on the diets of omnivorous or insectivorous mammals. Many omnivorous species rely on these resources seasonally, feeding on higher-quality arthropods, fungi, nuts, and seeds in warmer months, while using cached nuts and low-quality plant material (e.g., bark, roots) in winter (del Valle and Busch 2003). Beyond resource abundances, the toxicity of some plant compounds is temperature-dependent and can be exacerbated with global change, which may impact the fitness of herbivores (Dearing 2013). Finnish reindeer (*Rangifer*) herders have reported an increase in mold growth on pastures due to unfrozen soil, and some of the most abundant fungi produce mycotoxins, which can cause a variety of symptoms (e.g., gastrointestinal distress, reduced body condition, behavior issues) and even death in some instances (Turunen et al. 2009; Kumpula et al. 2024). Higher temperatures coupled with lower precipitation can also increase tannin levels (Monteiro et al. 2006), which can decrease the digestibility of plants (Robbins et al. 1991; Bozinovic, Novoa, and Sabat 1997). The wide implied range of dietary responses to global change makes this a key target for future research.

### Macroscopic ITV

For mammals that incorporate substantial amounts of plants in their diet (herbivores and omnivores), the hindgut sections responsible for processing this material (i.e., cecum and colon) should be the most responsive to changes in dietary quality. In Order Rodentia, this is supported in some studies (Hammond and Wunder 1991; Borkowska 1995; Campbell and MacArthur 1996), but not others (Derting and Hornung 2003; del Valle, Busch, and López Mañanes 2006; Derrickson 2013). However, all sections of mammalian GITs have been documented to respond when dietary quality is experimentally manipulated, which is most commonly done by increasing fiber content (e.g., Addis 1932; Loeb, Schwab, and Demment 1991; Lee and Houston 1993; Pei, Wang, and Hume 2001). Aside from fiber, high -fat and high-sugar diets have also been shown to cause increases in the GIT size of Yunnan red-backed voles (*Eothenomys eleusis*; Geng, Zhu, and Yang 2024), possibly via disruption of the gut microbiome (Do et al. 2018) and onset of inflammation leading to increased epithelial cell proliferation and intestinal thickness (Malesza et al. 2021; Papoutsis et al. 2022). Digesta particle size may also induce GIT ITV; tammar wallabies (*Notamacropus eugenii*) maintained on a diet of finely ground pellets had longer and heavier intestines (Munn, Banks, and Hume 2006) and longer ceca (Munn et al. 2009) than those on a diet of coarse hay, although fiber content was similar. While low-quality, high-fiber diets usually mean larger digesta particles, a higher level of smaller particles could increase the viscosity of digesta, causing a reduction in absorptive capabilities and a lengthening of the gut (Munn, Banks, and Hume 2006). Thus, increases in fiber, fat, sugar, and digesta viscosity often result in hypertrophy of some or all sections of the GIT across a wide variety of mammal species.

### Microstructural ITV

Diet quality can also impact gut microstructural aspects such as villus dimensions and crypt depth. Bujard (1908) manipulated the diets of *Rattus norvegicus*, finding that a diet of milk or animal matter resulted in SI villi that were tall and narrow, but shorter and wider when fed plant matter or a combination of milk and cellulose. The shorter and broader villi may serve to slow the passage of digesta (Barry 1976), while also maximizing contact of the mucosa with foodstuff. Similarly, swamp wallabies (*Wallabia bicolor*) and domestic rabbits (*Oryctolagus cuniculus*) consuming higher fiber diets had shorter villi (Osawa and Woodall 1990; Chiou, Yu, and Lin 1994). Studies on Norway rats (*R. norvegicus*) and domestic pigs (*Sus scrofa*) have also shown that higher levels of protein lead to deeper crypts in the SI (Hadžiomerovíc et al. 2025; Zeng et al. 2025). Deeper intestinal crypts are usually associated with decreased intestinal health and digestive efficiency, and excessive levels of protein can decrease gut health by altering the gut microbiome and damaging the intestinal lining (Ma et al. 2017).

The weaning period, which is when mammals begin to incorporate solid sources of nutrition beyond milk, also marks a significant shift in dietary composition that can reshape the gut microstructure (Sakata and Setoyama 1997). Studies on piglets (*Sus*) have found that crypt depth and villus complexity increase during weaning while villus height decreases, changes that can be associated with decreased gut health (Hampson 1986; Nabuurs et al. 1993). However, individuals given supplemental nutrition during this time were protected against these changes (Nabuurs et al. 1993; Pluske, Williams, and Aherne 1996).

### Physiological ITV

Poor dietary quality (e.g., high fiber, increased tannins) can decrease digestive efficiency due to dysbiosis of the microbiome (De Marco et al. 2021), inflammation of the intestinal lining (Nogueira et al. 2022), or altered transit time (Kashyap et al. 2013). One way that digestive efficiency is altered is via changes in digestive enzyme activity. Multiple studies have examined how diet changes such as increased fiber or altered protein, fat, and/or starch levels impact digestive enzyme activity; these changes can cause altered small intestinal sucrase (Galluser, Raul, and Canguilhem 1988; Bozinovic, Novoa, and Sabat 1997), maltase (Sabat, Bozinovic, and Zambrano 1995), protease (necessary for protein digestion; del Valle, Busch, and López Mañanes 2006), and aminopeptidase-N (Sabat and Bozinovic 2000; del Valle and López Mañanes 2008; Cortés et al. 2011) activity. Shifts in diet have more recently been shown to alter the composition of gut microbial and fungal communities (Cai et al. 2019; Bo et al. 2020; Barts et al. 2025), including responses that improve the efficiency of digestion. This can be done through changes in bacterial functional groups; for example, when montane voles (*Microtus montanus*) were given a higher fiber diet, bacteria associated with fiber fermentation tended to increase (Barts et al. 2025).

As stated previously, studies that integrate morphological and physiological traits are necessary to advance mechanistic knowledge of GIT ITV. In *R. norvegicus*, individuals fed fiber in the form of soluble polysaccharides compared to insoluble cellulose had a higher rate of mucosal cell proliferation (Johnson and Gee 1986). *Mus musculus* given a high-fat diet showed a stress response in crypt cells as well as increased intestinal proliferation after only one day, a way for the intestinal epithelium to maximize fat absorption (Enriquez et al. 2022). Specifically, the enterocytes within the jejunum were functionally altered to become more efficient at lipid absorption (Enriquez et al. 2022). Ultimately, we expect altered dietary quality to affect multiple levels of GIT morphology and function as individuals seek to maximize digestive efficiency and nutrient absorption.

### Hibernation, starvation, and fasting

GIT ITV in response to hibernation or food deprivation has been observed in some or all sections of GITs from three mammalian Orders (Microbiotheria, Rodentia, and Scandentia, e.g.,Gao et al. 2016; Jia et al. 2025; Sabat et al. 2025). As endotherms, mammals must either maintain high body temperature or utilize strategies for controlled temperature reduction, usually in response to seasonal cycles. While many mammals employ torpor (i.e., reduced metabolic rate for periods up to 24 h) to conserve energy, relatively few species are obligate hibernators. Utilization of torpor could minimize the seasonal plasticity required of the GIT, but any observation of torpor is likely indicative of energetic limitation and thus it may be difficult to isolate how torpor and gut plasticity interact in isolation. Indeed, Piscitiello et al. (2021) manipulated photoperiod to induce bouts of torpor in Djungarian hamsters (*Cricetiscus sungorus*), finding that increased photoperiod-induced torpor was associated with longer SIs. Though rarer as an energetic strategy, hibernation has been more commonly studied than torpor with respect to GIT ITV.

The gut of many hibernating mammals atrophies during hibernation (Galluser, Raul, and Canguilhem 1988; Carrey 1990; Hume et al. 2002; Zheng et al. 2024), which involves significant remodeling at various levels (e.g., cellular, organ, and whole organism; Kurtz et al. 2021). Following hibernation, individuals are typically triggered to emerge by spring temperatures, and warmer winters have been shown to result in animals such as marmots (*Marmota*) emerging several weeks earlier (Inouye et al. 2000; Bonenfant et al. 2026), which can result in mismatches between emergence and food availability (Cordes et al. 2020). Earlier emergence from hibernation could result in phenological mismatches between what food resources are necessary for an animal to make these extreme GIT changes, and what is actually available on the landscape.

Climate change can also exacerbate and accelerate the spread of diseases that hibernators are susceptible to, such as white-nose syndrome, a fungus (*Pseudogymnoascus destructans*) that has decimated hibernating bat populations across North America (Reeder et al. 2012). The fungus increases the number of times that bats arouse from hibernation, which rapidly depletes their fat stores and can lead to death by starvation (Reeder et al. 2012). Since these changes are intimately associated with energetics, it will be critical to extend these studies to understand gut structure and function.

### Macroscopic ITV

The GIT is one of the most expensive tissues for an individual to maintain (Aiello and Wheeler 1995; Rolfe and Brown 1997). During hibernation, the GIT atrophication is part of a controlled reduction in metabolism. Hume et al. (2002) examined the GITs of wild alpine marmots (*Marmota marmota*) following emergence from hibernation, finding that various sections of the gut became longer and heavier post-hibernation. This same pattern was also seen in Daurian ground squirrels (*Spermophilus dauricus;*Zheng et al. 2024).

Although hibernation involves a drastic reduction in the amount of food an individual takes in, starvation and fasting represent a different energetic strain on an individual, and induces an acute stress response, rather than controlled changes. Several lab studies have tested how starvation and/or fasting influences the gut independent of hibernation. Some work has found that rodent GITs become lighter during starvation (Thaysen and Thaysen 1949; Kristan and Hammond 2001), while others find heavier and/or longer gut sections (Zhao and Cao 2009; Gao et al. 2016). This suggests that while starvation and fasting *can* cause atrophy of the gut, some species may instead adjust their GIT morphology during periods of low food in order to increase retention time. Responses of the GIT to starvation and fasting are variable and species-specific, while changes in the GIT before, during, and after hibernation are largely predictable.

### Microstructural ITV

The atrophication (during hibernation) and remodeling (post-hibernation) of the GIT involve clear microstructural changes of the mucosa and villi. After emergence, epithelial cell proliferation must increase and villi in the SI must strengthen in order for nutrients to be efficiently absorbed. Mucosal mass of European hamsters (*Cricetus cricetus*) decreased during hibernation (Galluser, Raul, and Canguilhem 1988), and hibernating thirteen-lined ground squirrels (*Ictidomys tridecemlineatus*) who underwent a jejunal bypass showed decreased mucosal mass, villus height, and surface area in the bypassed sections, indicating that contact of the mucosa with foodstuff is the main factor that maintains mucosal mass during normal activity (Carey and Cooke 1991). Villus height and mucosal mass are also lower in other species of hibernating squirrels (Carey 1990; Zheng et al. 2024). Post-emergence, marmots showed a gradual increase in mucosal thickness throughout the gut (Hume et al. 2002). Fasting and starvation have also been shown to cause epithelial damage (Thaysen and Thaysen 1949), shorter villi, deeper crypts, and reduced epithelial turnover (Dou et al. 2001) in lab rats, but these changes were reversed after re-feeding. Overall, a reduction of foodstuff passing through the GIT, whether it be due to hibernation, starvation, or fasting, typically results in atrophication of GIT microstructure.

### Physiological ITV

As the GIT atrophies during hibernation, gut physiological processes are also altered. Galluser, Raul, and Canguilhem (1988) found that small intestinal lactase and aminopeptidase-N activity increased during hibernation in *C. cricetus*. In contrast, Sabat et al. (2025) showed that hibernating monitos del monte (*Dromiciops gliroides*) reduced intestinal sucrase, maltase, and protease activity as a way of conserving energy. Caloric restriction in mice has been shown to cause increased glucose transport capacity (Kristan and Hammond 2001) and sucrase activity (Zarling and Mobarhan 1987). It has also been shown that seasonal restructuring occurs in the gut microbiome, particularly in hibernators. During hibernation, the lack of foodstuff passing through the GIT results in less support for the growth of microbes in the gut (Carey, Walters, and Knight 2013), meaning microbial diversity and abundance must rapidly increase once food intake begins again after emergence. Microbial activity is typically lower immediately post-emergence (Hume et al. 2002), and microbial diversity lowest during hibernation (Carey, Walters, and Knight 2013; Dill-McFarland et al. 2014; Stevenson, Duddleston and Buck 2014). Microbial activity may also be affected by fasting; studies on small rodents have found that intermittent fasting can reduce the total bacteria present in some gut sections, as well as shifting the general composition of the gut microbiome (Jia et al. 2025). More work is needed to fully understand how the physiology of the gut and digestion are impacted by decreased levels of foodstuff within the GIT.

### Parasitic infection

GIT ITV in response to parasitic infection has only been observed in two Orders of placental mammals (Artiodactyla and Rodentia, e.g., Argenzio et al. 1990; Kristan and Hammond 2001). Infection response usually involves energetic costs such as maintenance and growth of tissues towards successful avoidance of infection. Wild mammals are susceptible to a wide variety of external and internal macro- and microparasites, which may interfere in various ways with energy allocation, but we focus here on intestinal parasites. Chronic infections of helminths (phyla Nematoda and Platyhelminthes), parasitic worms that inhabit the GIT, have negative consequences on the energetics of their hosts (Shanebeck et al. 2022), which must find ways to mediate these costs. As global change alters species ranges, host-parasite interfaces may shift to include novel intestinal parasites (Polley, Hoberg, and Kutz 2010; Okulewicz 2017), especially because the development of intestinal parasites is tightly correlated with climatic conditions (Kutz et al. 2004). For example, the hookworm *Ancylostoma braziliense* is endemic to the tropics but has begun to infect people living in Southern Europe (García-Rodrigo, Romero, and Olivo 2017; Okulewicz 2017), a result of the warming climate. *Setaria tundra* is a nematode parasite that was first discovered in Russian reindeer (*R. tarandus*), but has since spread across Europe, where it is attributed to the deaths of thousands of European reindeer (Enemark et al. 2017; Okulewicz 2017; Oloś, Nowakowska and Welc-Falęciak 2021).

### Macroscopic ITV

One way that hosts may buffer against the energetic cost of endoparasitic infections is by lengthening the GIT to maximize their own nutrient absorption. Raines (1989) experimentally infected captive prairie voles (*Microtus ochrogaster*) with the protozoan intestinal parasite *Eimeria ochrogasteri* and maintained them at two different temperatures. While not a helminth, animals infected with *E. ochrogasteri* had heavier intestines, but significantly shorter ceca. *Mus musculus* infected with the intestinal nematode *Heligmosomoides* likewise displayed longer and heavier SIs (Kristan and Hammond 2001; Kristan and Hammond 2003; Kristan and Hammond 2004) and heavier ceca (Kristan and Hammond 2001). Kristan and Hammond (2004) also examined the effects of parasitic infection duration, finding that stomach wet mass decreased with longer infections and SI dry mass increased with infection duration. While research on this stressor is limited, parasitic infections in rodents typically cause size increases in some or all sections of the GIT.

### Microstructural ITV

As with other stressors, such changes in GIT macrostructure often derive from microstructural changes. Kristan and Hammond (2003; 2004) also found that *H. polygyrus* infection alters mucosal mass in *M. musculus*, leading to more mucosal and serosal tissue in the SI (Kristan and Hammond 2003). A later experiment found that both mucosal and serosal tissue mass were significantly correlated with infection duration as well (Kristan and Hammond 2004). Possible explanations for this increase include compensation for decreased digestive efficiency among infected individuals (Kristan and Hammond 2003), although accumulation of larvae that did not emerge into the lumen of the intestine before weighing could not be ruled out (Kristan and Hammond 2004). Changes in serosal mass may also indicate altered peristalsis (the contractions of the smooth muscle that push foodstuff through the GIT) as a result of parasitic infections (Kristan and Hammond 2004). Piglets (*Sus*) experimentally infected with *Cryptosporidium parvum* (a microparasite that causes severe diarrhea and malabsorption) showed decreased villus height, increased crypt depth, and an overall reduction in the epithelial area (Argenzio et al. 1990), indicating parasitic infections also alter the gut microstructure of these two study systems.

### Physiological ITV

Endoparasites are extremely common in humans, with 24% of the globe estimated to have an active intestinal parasitic infection (Ahmed 2023). These infections can cause significant gastrointestinal issues such as dysbiosis, inflammation, chronic diarrhea, and malabsorption. However, minimal research has been done on how parasitic infections influence digestive physiology in nonhuman mammals, though some experiments indicate infection causes diminished uptake of glucose. Kristan and Hammond (2001) show that parasitized *M. musculus* have lower glucose uptake capacity and uptake rate, and lower glucose uptake rate was also found by Kristan and Hammond (2003), although infection did not significantly affect glucose uptake capacity. *Sus* piglets with cryptosporidiosis had impaired glucose-stimulated sodium and water absorption in the jejunum and ileum several days after infection (Argenzio et al. 1990). Wild mammals have been shown to have higher rates of endoparasitic infections relative to humans (over 60% in some studies; Liatis et al. 2017; Rahman et al. 2018; Riebold et al. 2020; Ferdous et al. 2023), so more work needs to be done to determine to what degree infections alter GIT morphology and digestion.

### Seasonality

Many of the stressors discussed above vary (or, co-vary) naturally within the lifetimes of individual mammals. Not all studies have isolated the effects of single stressors; nevertheless, an important literature exists that we organize around the concept of “seasonality” here. Seasonal ITV has been observed in some or all sections of GITs from seven mammalian Orders (Artiodactyla, Carnivora, Lagomorpha, Macroscelidea, Microbiotheria, Rodentia, and Scandentia, e.g., Staaland, Jacobsen, and White 1979; Hammond 1993; Derting and Noakes 1995; Schwaibold and Pillay 2003; Bełżecki et al. 2017; Chapman and McLean 2023). Much of this ITV may be a response to seasonal reproductive cycles, which are organized around resource pulses in many mammals (e.g., those with long life spans or at higher latitudes) and may be cued by photoperiod (Bronson 2009). Therefore, global changes in seasonality may be expected to affect the magnitude of GIT plasticity vis-à-vis reproduction and the energy it requires. Mammals that use photoperiod as a cue for breeding may actually be more susceptible to trophic mismatches; for example, caribou (*Rangifer tarandus*) use this cue to begin migration to where calves will be born, but warming temperatures is causing an earlier onset of the peak resource pulse. This mismatch has resulted in rising caribou offspring mortality in the Arctic (Post and Forchhammer 2008).

### Macroscopic ITV

A majority of GIT studies spanning seasons report increased length or mass in one or more sections of the colder months (e.g., Hammond 1993; Derting and Hornung 2003; Chapman and McLean 2023), although other studies found no seasonal patterns in these metrics (McDevitt and Speakman 1994; Voltura 1997). Change during colder months is often coincident with decreases in dietary quality (i.e., increased plant material; Bozinovic, Novoa, and Veloso 1990; Chapman and McLean 2023) and lengthening is expected to increase retention time and maximize nutrient absorption, although in this case the effects of season and diet are confounded. However, Mulvey et al. (2024) found that striped mice (*Rhabdomys pumilio*) in South Africa had heavier SIs during the wet season, when food availability was more than double that of the dry season (Mulvey et al. 2024), suggesting that seasonal trends are species- and location-specific and may not be directly related to temperature.

### Microstructural ITV

Seasonal changes have also been observed in the GIT microstructure of small mammals. Borkowska (1995) examined seasonal changes in the mucosal surface of the striped field mouse (* Apodemus agrarius*). Villus surface area as well as density (i.e., villi/mm^2^) was lowest in late fall, potentially because this period of time was the least stressful period throughout the year due to the increased animal material in diets and reduced need for absorptive area to meet energetic demand. Much more work is needed to uncover the proximate factors that drive seasonal GIT ITV across mammals.

### Physiological ITV

Very little work has been done on how seasonality, independent of hibernation, alters the physiology of mammal GITs. Galluser, Raul, and Canguilhem (1988) monitored the response of several digestive enzymes to changing seasons and diets in the European hamster (*C. cricetus*). While deep hibernation significantly altered lactase (which breaks down lactose) and aminopeptidase-N activity, season itself did not have an effect on the intestinal enzymes in this controlled environment outside the hibernation window. Again, more work must be done to uncover functional shifts in the GIT of mammals across annual cycles characterized by multiple, potentially covarying factors.

### Conclusions and future work

**Fig. 4:**
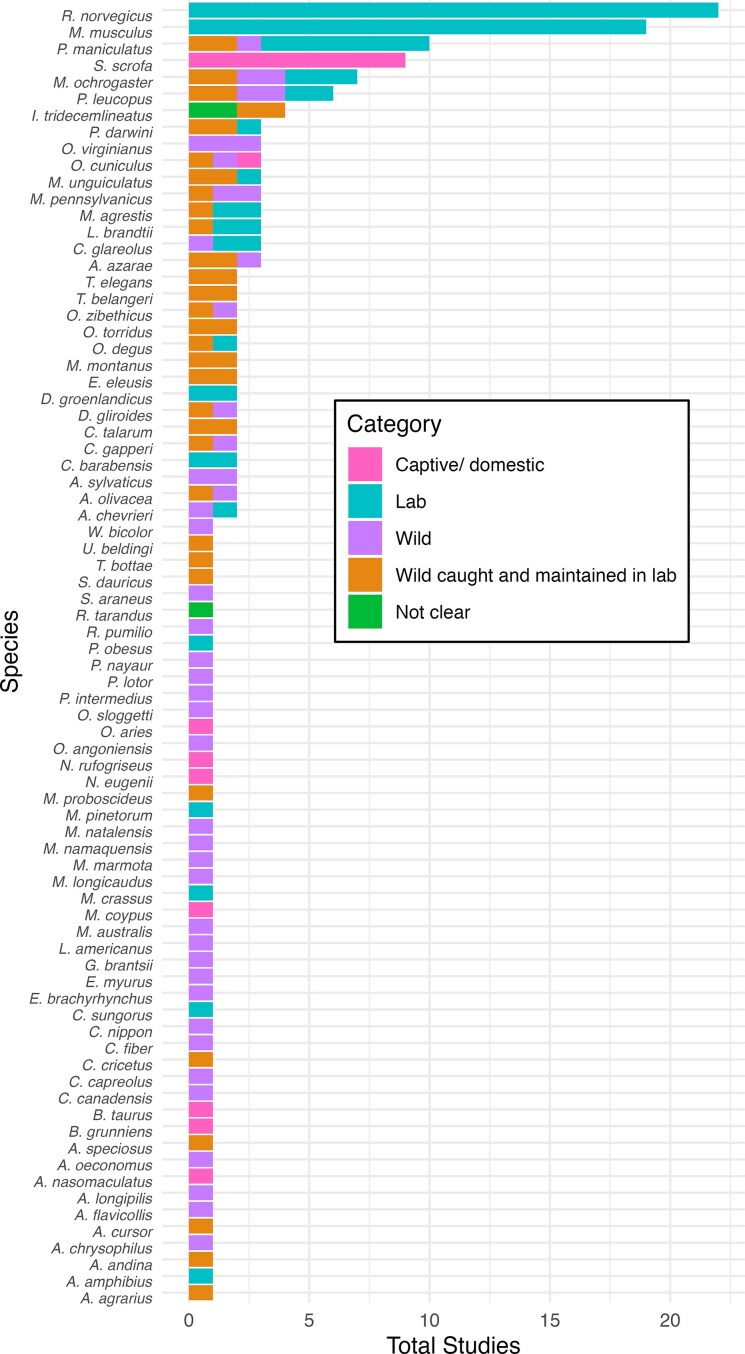
Barplot of all studies of mammalian gastrointestinal tract intraspecific variation in our dataset, grouped by species and colored by whether the study was done on domestic animals, lab animals, wild animals, wild animals maintained for a period of time in a lab, or the source was unclear.

A century of basic research on gastrointestinal tract ITV (Fig. 2, Supplementary Material 2) has provided a powerful foundation for understanding how mammals utilize this phenomenon to persist in dynamic environments. While we have attempted to be exhaustive, it is possible that some sources were missed in our literature review. Nevertheless, it is to our knowledge the most comprehensive collection of papers quantifying the number and identity of mammalian species for which GITs have been measured to date and, in particular, those that have explored mammalian GIT ITV. Additionally, while most studies have been on lab or captive populations (Fig. 4), we have attempted to reorganize knowledge of responses to certain treatment types around environmental and ecological variables where global-scale changes are already occurring or likely to occur. However, numerous shortfalls still limit understanding of how GIT ITV will, or will not, buffer mammals in the wide variety of habitats in which they occur. First, as stated above, most work has been done in lab rats and mice in controlled settings (Fig. 4). The three best-studied species in the context of GIT ITV are *R. norvegicus, M. musculus*, and *Peromyscus maniculatus*; out of a combined 51 studies on these species, only one was done on wild individuals not maintained in a lab prior to euthanization (Chapman and McLean 2023). Responses of GITs to varying energetic demands may be far more informative about functional responses when conducted in a natural setting. Individuals raised and maintained in captivity have increased rates of reproduction and larger litter sizes (Derting and McClure 1989), which may overestimate energy demand and result in more extreme morphological changes (Derting and Hornung 2003). Concurrent gestation and lactation are common in lab populations of mice, but it is unclear to what degree this happens in wild mice (Wilsterman and Cunningham 2022). Of the few studies in wild individuals (Myrcha 1964; Myrcha 1965; Gębczyńska and Gębczyński 1971), smaller magnitudes of ITV are observed than what has been shown in lab animals (Speakman 2008). Therefore, renewed focus should be placed on study of GIT ITV in animals captured in the field, ideally in conjunction with collection of high-resolution dietary and environmental data, in order to improve understanding of how wild individuals cope with energetic stressors being altered through global change.

Second, we found that existing research is highly uneven across the mammalian tree of life (Fig. 3; Table 1). This is a problem because reproduction (a major energetic demand that stimulates GIT ITV) varies substantially across higher mammalian clades. In regard to reproduction, the cost of lactation is high for all mammals (Stannard, Miller, and Old 2020), although the length of lactation and thus its total cost varies among species. The majority of research on mammalian GIT ITV has been on placentals (93.6% of mammal species examined for GIT ITV are placentals), with most work done on GIT ITV in rodents, but moving beyond this group may reveal different magnitudes of ITV. A study on common shrews (*Sorex araneus*), a eulipotyphlan species which has been extensively studied regarding ITV in tissues other than the GIT (Taylor, Baldoni and Dechmann 2026), found reproduction led to longer GITs and more villi, meaning an increased absorptive area (Jaroszewska and Wilczyńska 2006). Similarly, lactation resulted in racoons (*Procyon lotor*, Order Carnivora) having longer and heavier guts (Derting 1996). Several studies have examined the response of GITs to reproduction in large artiodactyls (sheep, cows, and deer), with most showing similar trends as seen in rodents (Fell, Campbell, and Boyne 1964; Smith and Baldwin 1974; Jenks et al. 1994; Jiang et al. 2009), but a broader comparative approach targeting poorly represented Orders of mammals is essential. For example, bats (Order Chiroptera) have never been explored in the context of GIT ITV, but their highly specialized mode of locomotion (flight) have resulted in unique reproductive trade-offs that could have unusual impacts on GIT ITV (Barclay 1994; Culina et al. 2019). Monotremes have also never been tested for GIT ITV, but their highly distinct reproductive strategy may also result in unique responses of the gut to lactation.

**Table 1:** The 27 mammalian orders currently recognized by the Mammal Diversity Database, with the total number of living species within each. “Measured for GIT traits” shows the number and percentage of species within each Order that have any type of quantitative GIT trait available, while “Examined for GIT ITV” shows the number and percentage of species within each Order that have been examined for GIT ITV.

Order	Total living species	Species in Order measured for GIT traits		Species in Order examined for GIT ITV	
Monotremata	5	2	40%	0	0%
Paucituberculata	7	0	0%	0	0%
Didelphimorphia	128	15	11.7%	1	0.8%
Microbiotheria	2	1	50%	1	50%
Notoryctemorphia	2	0	0%	0	0%
Dasyuromorphia	87	4	4.6%	0	0%
Peramelemorphia	23	2	8.7%	0	0%
Diprotodontia	151	29	19.2%	3	2%
Cingulata	25	1	4%	0	0%
Pilosa	17	4	23.5%	0	0%
Macroscelidea	20	7	35%	3	15%
Afrosoricida	55	11	20%	0	0%
Tubulidentata	1	0	0%	0	0%
Proboscidea	3	2	66.7%	0	0%
Hyracoidea	6	4	66.7%	0	0%
Sirenia	4	2	50%	0	0%
Dermoptera	2	0	0%	0	0%
Scandentia	23	7	30.4%	1	4.3%
Primates	512	96	18.8%	0	0%
Lagomorpha	113	13	11.5%	2	1.8%
Rodentia	2733	319	11.7%	56	2%
Eulipotyphla	605	33	5.5%	1	0.2%
Chiroptera	1492	78	5.2%	0	0%
Pholidota	9	4	44.4%	0	0%
Carnivora	313	78	24.9%	1	0.3%
Perissodactyla	18	10	55.6%	0	0%
Artiodactyla	367	100	27.2%	10	2.7%

Third, we must expand our concept of GIT ITV to finer levels and functions. Although we discuss traits broadly here (including physiological measures), most existing data are from measurements of macroscopic and microscopic morphology. Moreover, the most commonly used macroscopic traits discussed may be too coarse. The SI has traditionally been divided into three functional regions of approximately equal sizes; the proximal, medial, and distal region, defined as the duodenum, jejunum, and ileum (Fell, Smith, and Campbell 1963; Cortés et al. 2011; Zhang et al. 2016; Wang, Caviedes-Vidal, and Karasov 2019; Zwick et al. 2024; Hadžiomerovíc et al. 2025). These three regions have loosely defined boundaries (Brambell 1965) and are understood to play different roles in digestion, but new genetic work has identified five distinct functional regions of both human and mice SIs (Zwick et al. 2023). This suggests that current morphological delineations may map poorly to functional regions. Future work should aim to incorporate these new functional regions in work on GIT ITV. In addition, the role of enzymes and microbiome in the role of GIT ITV is a developing field, but both are clearly important in response to changing energetic demands (Karasov and Hume 1997; Karasov and Douglas 2013). It is well known that an individual’s gut microbiome is a major factor relating to the health and wellness of that organism (Holmes et al. 2011; Tuddenham and Sears 2015; Gould et al. 2018), which warrants more research into how seasonal shifts in this trait could mediate stress response. It is important to note that the microbiomes of laboratory mice differ significantly from the microbiomes of wild mice (Weldon et al. 2015; Rosshart et al. 2017), so future studies should prioritize work on wild individuals to understand the natural response of small mammal’s gut microbiomes to changing energetic demands.

Key opportunities also exist to study the GIT response to new and emergent environmental changes. For example, microplastics are ubiquitous in most environments (Loganathan and Kizhakedathil 2023) and can assimilate into the body via ingestion, inhalation, and skin contact (Wu et al. 2022). Microplastics have been found in the GITs of fishes (Zazouli et al. 2022), dolphins (Battaglia, Beckingham, and McFee 2020), shrimp (Saborowski, Paulischkis, and Gutow 2019), bats (Correia et al. 2023), birds (Carlin et al. 2020; Tarricone et al. 2024), and humans (Jones, Wright, and Gant 2023). These exogenous particles may have fitness consequences and induce GIT response under certain conditions. A 2022 study on experimentally introduced microplastics in African clawed frogs (*Xenopus laevis*) found that larvae exposed to either microplastics or cellulose exhibited longer and heavier GITs (Ruthsatz et al. 2022), suggesting that the body responds to microplastics in a similar fashion as low-quality coarse fibrous digesta. However, microplastics are completely indigestible (Saborowski, Paulischkis, and Gutow 2019) so increases gut size will not lead to increased nutrient absorption; conversely, the energetic cost of maintaining a larger GIT could decrease fitness. Microplastics are known to cause intestinal damage and inflammation in lab mice (Jia et al. 2023), but we are not aware of any studies on microplastics and plasticity of GIT morphology in mammals.

Finally, developing more comprehensive databases of organismal trait data and associated ontologies is necessary to expedite all of the research above. The still-laborious process of collecting data for literature reviews like ours highlights the need for future GIT traits to be digital, recorded at the individual (as opposed to species or higher taxon) level, accessible on some type of public data repository, and mappable to museum specimens (McLean et al. 2025), zoo animals (Poo et al. 2022), or lab strains wherever possible. We found many studies of rare GIT traits or taxa from supplemental files of publications that are difficult and time-consuming to locate and synthesize. More critically, some authors did not publish the raw GIT measurements they report. All of these best practices will open new frontiers to understand how wild species utilize GIT ITV as a buffer in changing environments.

## Supplementary Material

icag070_Supplemental_Files ** Supplementary Figure 1**. Phylogeny of mammals pruned to those species with quantitative macroscopic or microstructural gastrointestinal tract traits available, colored by Order. Black bars represent the number of studies done on each species. Scale bar shows the height of 33 studies, which was the highest number of studies done on any one species. If any study exists in a later meta-analysis or review, it appears in this figure twice.

## Data Availability

The data underlying this article are available in the article as well as in the online supplementary material.
